# Renal Intercalated Cells Sense and Mediate Inflammation via the P2Y_14_ Receptor

**DOI:** 10.1371/journal.pone.0121419

**Published:** 2015-03-23

**Authors:** Anie Azroyan, Virna Cortez-Retamozo, Richard Bouley, Rachel Liberman, Ye Chun Ruan, Evgeny Kiselev, Kenneth A. Jacobson, Mikael J. Pittet, Dennis Brown, Sylvie Breton

**Affiliations:** 1 Center for Systems Biology, Massachusetts General Hospital/Harvard Medical School, Boston, Massachusetts, United States of America; 2 Program in Membrane Biology/Nephrology Division, Massachusetts General Hospital/Harvard Medical School, Boston, Massachusetts, United States of America; 3 Laboratory of Bioorganic Chemistry, National Institute of Diabetes and Digestive and Kidney Diseases, National Institutes of Health, Bethesda, Maryland, United States of America; Universidade de Sao Paulo, BRAZIL

## Abstract

Uncontrolled inflammation is one of the leading causes of kidney failure. Pro-inflammatory responses can occur in the absence of infection, a process called sterile inflammation. Here we show that the purinergic receptor P2Y_14_ (GPR105) is specifically and highly expressed in collecting duct intercalated cells (ICs) and mediates sterile inflammation in the kidney. P2Y_14_ is activated by UDP-glucose, a damage-associated molecular pattern molecule (DAMP) released by injured cells. We found that UDP-glucose increases pro-inflammatory chemokine expression in ICs as well as MDCK-C11 cells, and UDP-glucose activates the MEK1/2-ERK1/2 pathway in MDCK-C11 cells. These effects were prevented following inhibition of P2Y_14_ with the small molecule PPTN. Tail vein injection of mice with UDP-glucose induced the recruitment of neutrophils to the renal medulla. This study identifies ICs as novel sensors, mediators and effectors of inflammation in the kidney via P2Y_14_.

## Introduction

Kidney failure is almost always associated with uncontrolled inflammation [1,2]. A link between renal inflammation and signaling via purinergic receptors has been established, but very little is currently known about the underlying mechanisms involved and how to prevent and alleviate the severe damage cause by inflammation. Numerous purinergic receptors are expressed in the kidney, and deregulation of purinergic signaling is associated with several pathologies, including hypertension, chronic kidney disease, acute kidney injury, diabetic nephropathy and glomerulonephritis [3,4]. Purinergic receptors are involved in the regulation of water, electrolyte, and volume homeostasis by collecting duct principal cells [5–8]. However, there is limited knowledge on the purinergic regulation of the other major cell type of the collecting duct, the intercalated cell (IC). ICs participate in the maintenance of acid/base homeostasis via the proton-pumping V-ATPase [9,10]. In the epididymis, ATP and adenosine are potent activators of V-ATPase-dependent proton secretion in clear cells, which are analogous to ICs [11]. Extracellular ATP stimulates bone resorption in osteoclasts, a process that also requires activity of the V-ATPase [12,13]. These studies suggest a role for the purinergic regulation of acid/base transport in the kidney, but the purinergic receptor signature of ICs still remains to be characterized.

Nucleotide-activated purinergic receptors are separated into two families, P2X receptors that are ligand-gated ion channels, and P2Y receptors that are G protein-coupled receptors (GPCRs) [5–7]. Based on its homology with other P2 receptors, p2y5 was initially proposed to be a nucleotide-receptor, but it was subsequently shown to be insensitive to nucleotides [14] and to bind lysophosphatidic acid (LPA) [15]. Similarly, p2y10 is a lysophospholipid receptor that is not activated by nucleotides [16]. The P2Y_14_ receptor (also known as GPR105) is the most recent addition to the P2Y receptor family [17,18]. P2Y_14_ is specifically activated by nucleotide sugars including UDP-glucose and it is insensitive to ADP/ATP and UTP [18]. While UDP-glucose is used in the metabolism of nucleotide sugars, it is also released by cells and acts as an autocrine activator of the P2Y_14_ receptor. Most nucleotides are rapidly degraded by ectonucleotidases after their release, but UDP-glucose resists hydrolysis by these enzymes [19]. While virtually all cells release nucleotides under basal conditions [20], this release can be accentuated in response to stimuli leading to activation of purinergic receptors [21]. UDP-glucose, extracellular ATP and adenosine are emerging as immune-regulatory factors known as DAMPs (damage associated molecular pattern) molecules [22,23]. DAMPs initiate sterile inflammatory reactions, as opposed to PAMP (pathogen associated molecular patterns), which perpetuate infectious pro-inflammatory responses [23–26].

The role of P2Y_14_ as an inflammatory mediator was suggested based on its high expression levels in immune cells, and on the increased release of its ligand, UDP-glucose, by damaged cells [17,20,27–32]. In addition to immune cells, several tissues including the brain, the gastro-intestinal tract, the kidney and the lung express P2Y_14_ mRNA [18]. Patients with cystic fibrosis and asthma secrete high amounts of UDP-glucose in their lungs [27,33], and P2Y_14_ activation by UDP-glucose in airway epithelial cells leads to IL-8 secretion [34]. Furthermore, injection of UDP-glucose into the mouse uterus induces the recruitment of neutrophils into the endometrium [35]. Another indication of the inflammatory role of P2Y_14_ is the up-regulation of its mRNA expression by lipopolysaccharides (LPS) [35,36]. However, while purinergic signaling has been established in the modulation of renal inflammation, the role of P2Y_14_ in mediating immune responses in the kidney has not been described. The objective of this study was, therefore, to examine the potential participation of this receptor in the initiation of inflammation in the kidney.

In this study we uncover elevated expression of a restricted number of P2 receptors in ICs, most notably P2Y_14_. Moreover, P2Y_14_ expression was not detectable in other renal epithelial cells. We also provide evidence that P2Y_14_ activation by UDP-glucose induces a pro-inflammatory response in ICs that is mediated by activation of the MAPK pathway. This is followed by increased expression of pro-inflammatory chemokines in ICs, and subsequent neutrophil infiltration in the renal medulla. Thus, we have identified a novel inflammatory role for renal ICs via P2Y_14_ signaling.

## Materials and Methods

### Reagents and antibodies

Uridine 5′-diphosphoglucose disodium salt hydrate from *Saccharomyces cerevisiae (*UDP-glucose) and the MEK inhibitor PD98059 were purchased from Sigma Aldrich (St. Louis, MO). Uridine diphospho-D-[6-^3^H] glucose ([^3^H]UDP-glucose) was purchased from Perkin Elmer (Waltham, MA). PPTN, a selective high affinity antagonist of the P2Y_14_ receptor has been described previously [37]. The chicken antibody against the V-ATPase B1 subunit has been described previously [38]. An affinity-purified chicken antibody against the V-ATPase A subunit was raised against the same sequence as previously described for a rabbit anti-V-ATPase A subunit antibody [39,40]. A rabbit anti-P2Y_14_ antibody and its immunizing peptide were purchased from Alomone labs (Jerusalem, Israel). Rabbit anti-cytoskeletal actin antibody was from Bethyl Laboratory (Montgomery, TX). Rabbit anti-pendrin antibody was a kind gift from Dr. Aronson (Yale University). Rabbit monoclonal antibodies against p-p44/p42 MAPK and against total p44/p42 MAPK were purchased from Cell Signaling Technology (Boston, MA). Rat anti-Ly6G antibody was from Biolegend (San Diego, CA). Mouse anti-pan-actin was purchased from EMD Millipore (Billerica, MA). All secondary antibodies used were affinity purified and purchased from Jackson Immunoresearch (West Grove, PA), except for a goat anti-rat cy3, which was purchased from Invitrogen (Grand Island, NY). Streptavidin-fluorescein was purchased from Invitrogen. Cell culture medium was purchased from Invitrogen, and bovine serum was purchased from Atlanta Biologicals (Lawrenceville, CA).

### Animals

Adult male mice (eight to ten weeks old) were used for all experiments. Transgenic mice expressing EGFP under the promoter of the V-ATPase B1 subunit (B1-EGFP) mice have been described previously [41]. Wild type (C57BL/6 x CBAF1) mice were purchased from Jackson Laboratory (Bar Harbor, ME). Animals were housed under standard conditions and maintained on a standard rodent diet. The Massachusetts General Hospital (MGH) Subcommittee on Research Animal Care approved all animal studies, in accordance with National Institutes of Health, Department of Agriculture, and Accreditation of Laboratory Animal Care requirements. Our Institutional Animal Care and Use Committee (IACUC) specifically approved this study. For tail vein injections, animals were kept under isofluorane anesthesia (Baxter, Deerfield, IL) for several minutes to allow the injection of 200 μl of either saline solution or a saline solution containing 100 μM UDP-glucose (200 mg/kg of body weight).

### Isolation of intercalated cells from mouse kidneys

Mice were anesthetized using pentobarbital sodium (50 mg/kg body, ip, Nembutal, Abbott Laboratories, Abbott Park, IL). The blood was flushed out of the organs by perfusing the animals with a phosphate-buffered saline (PBS) through the cardiac left ventricle at a constant flow rate of 17 ml/min. Kidneys were excised and sliced, and some kidneys were microdissected to separate the cortex from the medulla. Tissues were then minced immediately in RPMI 1640 medium (Invitrogen, Grand Island, NY) containing 1.0 mg/ml collagenase type I (Invitrogen), 1.0 mg/ml collagenase type II (Sigma Aldrich) and 2 mg/ml hyaluronidase (Sigma Aldrich), and digested for 45 min at 37°C. A 40-μm-nylon mesh was used to remove undigested material following tissue digestion. Cells were then washed once with RPMI 1640 medium and once with a calcium-free PBS. EGFP-positive (EGFP(+)) and negative (EGFP(−)) cells were isolated immediately by FACS, based on their green fluorescence intensity, as we have described previously [42]. Cell isolation was performed at the MGH flow cytometry core facility using a modified FACS Vantage cell sorter (BD Biosciences, San Jose, CA). FACS isolated samples were used without delay for subsequent protein and RNA isolation.

### RNA isolation and RT-PCR

Total RNA was isolated from cells using RNeasy micro kit (Qiagen, Valencia, CA) and from tissues using RNeasy Mini kit, as we have described previously [42]. The sequences of the PCR primer sets, purchased from Invitrogen, are listed in Table 1. For end point PCRs, reaction mixtures consisted of a 20 μl final volume containing 2 μl template, 1.25 units AmpliTaq Gold DNA polymerase, 1× buffer II, 1.5 mM MgCl_2_, 1.0 mM each dNTP, and 0.5 μM forward and reverse oligonucleotide primers. The following parameters were used for PCR: 8 min at 95°C to activate the polymerase, 35 cycles of melting for 30 s at 95°C, annealing for 30 s at 60°C, extension for 30 s at 72°C, and a final extension for 10 min at 72°C. The amplification products were visualized by electrophoresis on a 1–2% agarose gel containing GelStar stain (Lonza, Rockland, ME). Real Time PCR was performed with a 7300 Real Time PCR system (Applied Biosystems). Amplification products were detected using the Power SYBR Green PCR master mix (Applied Biosystems), according to the manufacturer's instructions. Standard-curve relative quantifications were performed and relative values of each sample were normalized to GAPDH values. Samples were analyzed in triplicates for each experiment.

**Table 1:** Sequence of the primers used for RT-PCR detection.

**Gene**	**GeneBank accession**	**Forward primer (5'-3')**	**Reverse primer (5'-3')**	
			
Mouse *P2rx1*	NM_008771	AGGGTCTCAAACACCCACAG	AGCTGTTCCAACCCACAGAG
Mouse *P2rx2*	NM_153400	TTTGGCCCAACTTTGATCTC	CCCAGAGCAAGATGCCTATC
Mouse *P2rx3*	NM_145526	AGCCTCTTCTGGGACATCAA	GTTAGGGATGGCGCTGAGTA
Mouse *P2rx4*	NM_011026	AGCAGCTCTGTCCAAGCACT	TCCGAGGACAACTTCTCTGG
Mouse *P2rx5*	NM_033321	AAGGGGAGAGCAAACACTCA	CACCAACCAAACAGCAAGTG
Mouse *P2rx6*	NM_011028	GACGATCCTGGTCCAAGTGT	ACCAGGAACTCCAAGGGTTT
Mouse *P2rx7*	NM_011027	ACGCTTTCTTCAGCAGCAAT	GCTGCTCTCAGTTCTGACCA
Mouse *P2ry1*	NM_008772	GCATTTTGAGCCTTCTCAGG	CTCCCTCCAGCCAAACAATA
Mouse *P2ry2*	NM_008773	CAGCACAAACCATGCTGACT	ACAAGGGACCTCCTGTCCTT
Mouse *P2ry4*	NM_020621	CAGACCAAAGAGCACGAACA	GCTGGAACAGCAATGGAACT
Mouse *P2ry5*	BC069991	GGGCACTGAGAATTTTATCCA	GAGCAGTCCCAGTGGCTTAG
Mouse *P2ry6*	NM_183168	TAGGCCCTGGAATAGCAATG	TCTTGGCAAATGGATGTGAA
Mouse *P2ry10*	NM_172435	CAGAACCCCCATCTTCTCAA	GTGGTCCCTTCCTCTTCCTT
Mouse *P2ry12*	NM_027571	AAAATGCCTGCTGCTTGAAT	TGAAGAAATTCCAACAAAACGA
Mouse *P2ry13*	NM_028808	AAGCCACAGAGGCAAGAGAA	CCTGGAGTAAGGGACAGCAA
Mouse *P2ry14*	NM_133200	CCATGCAAAATGGAAGTCTG	CGGAAAGACTGGGTGTCTTC
Mouse *Gapdh*	NM_008084.2	GCACAGTCAAGGCCGAGAAT	GCCTTCTCCATGGTGGTGAA
Mouse *Slc26a4*	NM_011867.3	TTAGCAATGTTCGGATGTGC	GGCCAGCCTAACAGAGACAG
Mouse *Atp6v1b1*	NM_134157.2	ACACGGCGCTCTAAATCAGT	CCACCCACCTACACCAAAAG
Mouse *Slc4a1*	NM_011403.2	TAGAAATGAGGGCAGGGA	TGGCAAAACCTATTCCAA
Mouse *Cxcl1*	NM_008176.3	TGTTGTGCGAAAAGAAGTGC	CGAGACGAGACCAGGAGAAA
Mouse *Cxcl2*	NM_009140.2	CGGTCAAAAAGTTTGCCTTG	TCCAGGTCAGTTAGCCTTGC
Mouse *Tnf*	NM_013693.2	CCACCACGCTCTTCTGTCTAC	AGGGTCTGGGCCATAGAACT
Mouse *Ccl2*	NM_011333.3	CAAGAAGGAATGGGTCCAGA	AAGGCATCACAGTCCGAGTC
Mouse *Ccl3*	NM_011337.2	TAGCCACATCGAGGGACTCT	ACCAACTGGGAGGGAGATG
Mouse *Ccl4*	NM_013652.2	GATTTCCTGCCCCTCTTCTT	GGGAGACACGCGTCCTATAA
Mouse *Ccl5*	NM_013653.3	GTGCCCACGTCAAGGAGTAT	CCACTTCTTCTCTGGGTTGG
Mouse *Il1b*	NM_008361.3	GGGCCTCAAAGGAAAGAATC	TACCAGTTGGGGAACTCTGC
Mouse *Il6*	NM_031168.1	GTGGCTAAGGACCAAGACCA	ACCACAGTGAGGAATGTCCA
Canine *P2RX1*	XM_548344.2	TTCGCTTTGACATTCTCGTG	CATTTGCTCCGCATACTTGA
Canine *P2RX2*	XM_534633.3	ACACTCTCCATCCTGCTGCT	TGAGAGGAAGTCAGGGGAGA
Canine *P2RX3*	XM_540614.1	GACTGTCCTCTGCGACATCA	TTAGTGGCCGATGGAGTAGG
Canine *P2RX4*	XM_003639907.1	CCACAGTCCTCATCAGAGCA	GCCAGAGGTCACCTGAACAT
Canine *P2RX5*	XM_003639268.1	GCCAGCCACTTCTCTTTGTC	CAAATCCCACTCAGCCATTT
Canine *P2RX6*	XM_543562.1	CTGGGTGTGATCACCTTCCT	GAAGCTGGCTTTGTCTGCTC
Canine *P2RX7*	NM_001113456.1	CCCACATTAGGATGGTGGAC	CAGCCTGGACAAGTCTGTGA
Canine *P2RY1*	NM_001193673.1	GCTTGTGAAGAGGCAGGAAC	TCACTGGATCCACAGTCCAA
Canine *P2RY2*	XM_542321.2	CGTCAACGTGGCTTACAAGA	AATCCTCACTGCTGGTGGAC
Canine *P2RY4*	XM_003640257.1	ATGTGAGCTCTGGCAGCTTT	GGAAGCCACAGTGAGTGGAT
Canine *P2RY6*	XM_542320.1	CTTCCTGCCCTTCCATGTTA	GGCGGAACTTCTTCTGAGTG
Canine *P2RY10*	XM_549100.2	GCACTGCGGATGGTTTTTAT	CAGTGTGCTTTGGACAATGG
Canine *P2RY12*	NM_001003365.1	CAAGAGGCGTAGGCAAAGTC	GTAGGGAATGCGTGCAAAAT
Canine *P2RY14*	XM_542838.2	CTCATTACAGCTGCCGATCA	TCTAAAGGGCTGGCATAGGA
Canine *GAPDH*	NM_001003142.1	GCCCTCAATGACCACTTTGT	TCCTTGGAGGCCATGTAGAC
Canine *SLC26A4*	XM_540382.3	AACTCCGAGCTTCCAGTCAA	TCTCACTCCAACGACATCCA
Canine *ATP6V1B1*	XM_531858.3	CAAATCTACCCTCCGGTCAA	GGTTGATGAAGCTCCTCTCG
Canine *ATP6V0A4*	XM_539895.3	CAGCCTTGTCTTCAACGTCA	CTTGAGGTCGGTTCCCCTAT
Canine *SLC4A1*	NM_001048031.1	TCATCCTCACTGTGCCTCTG	CTCTGAGGCTCACACCTTCC
Canine *AQP2*	XM_543678.3	GGGCTCCCTCCTCTACAACT	GCAGCTCCACTGACTGTCG
Canine *IL8*	NM_001003200.1	TCAATTGAACCGCAATCCTA	TGCTTGTCGAGTTTTTGCTC
Canine *TNF*	NM_001003244.4	TCATCTTCTCGAACCCCAAG	CTGGTTGTCTGTCAGCTCCA
Canine *CCL2*	NM_001003297.1	CAAGAAAAGCCAAACCCAAA	GAGGGCATTTAGGGAAGGTT
Canine *CCL3*	NM_001005251.1	CAAGCCCGGTATTATCTTCG	AGGCTTTCAGCTTCAGATCG
Canine *CCL4*	NM_001005250.1	CTTTGAGACCAGCAGCCTCT	CAGTTCAGTTCCAGATCATCCA
Canone *CCL5*	NM_001003010.2	GCTCTGCAGTCAGGAAGGAG	GGCTGAGAGGATAGCTGTGG
Canine *IL1B*	NM_001037971.1	CCTGTGTGATGAAGGATGGA	TATATCCTGGCCACCTCTGG
Canine *IL6*	NM_001003301.1	CTCGGCAAAATCTCTGCACT	TGGAAGCATCCATCTTTTCC

### Flow cytometry analysis

Tissues and cells were prepared as described above for FACS. Prior to flow cytometry, cell suspensions were stained in PBS with BSA 1% using the following antibodies purchased from BD Biosciences (San Jose, CA): PE-conjugated anti-CD90 (clone 53–2.1), PE-conjugated anti-B220 (clone RA3-6B2), PE-conjugated anti-CD49b (clone DX5), PE-conjugated anti-NK1.1 (clone PK136), PE-conjugated anti-Ly-6G (clone 1A8), APC-Cy7-conjugated anti-CD11b (clone M1/70), PE-Cy7-conjugated anti-F4/80 (clone BM8), Alexa Fluor 700-conjugated anti-CD11c (clone HL3). Antibodies purchased from BD Pharmigen were also used: PE-conjugated anti-CD19 (clone 1D3), PE-Cy7-conjugated anti-B220 (clone RA3-6B2), FITC-conjugated anti-CD3e (clone 145-2C11), PE-Cy7-conjugated anti-CD4 (clone RM4-5) and PerCP-conjugated anti-CD8a (clone 53–6.7). Single cell suspensions were labeled for 45 min at 4°C. For monocyte/neutrophil staining, the following PE-conjugated antibodies were used: anti-CD90, anti-B220, anti-CD19, anti-CD49b, anti-NK1.1 and anti-Ly-6G. Neutrophils were defined as Lin+CD11b+ cells. B cells were defined as B220+CD19+ cells. Total T cells were defined as CD3e+ cells. CD4 T cells were defined as CD3e+CD4+ cells. CD8 T cells were defined as CD3e+CD8a+ cells. The number of neutrophils, B and T cells was defined as the total number of cells per organ multiplied by the percentage of each cell type identified by flow cytometry (LSRII; BD Biosciences). Cell suspensions obtained from the spleen were labeled with appropriate antibodies for staining controls. Data were analyzed with FlowJo v.8.8.7 (Tree Star, Inc., Ashland, OR).

### Cell culture and protein preparation

MDCK-C11 cells were cultured at 37°C in a 5% CO_2_–95% O_2_ mix in DMEM (Invitrogen) supplemented with 2 mM glutamine, 10% fetal bovine serum (Invitrogen), penicillin (100 U/ml), and streptomycin (100 μg/ml) (Invitrogen). Prior to any treatment, cells were serum starved for 24 hours. For cell surface biotinylation assays, cells were either grown to confluence on plastic dishes or on filters. Biotinylation was performed as described previously [43]. Proteins were subjected to SDS-PAGE following denaturation in Laemmli buffer for 5 min at 95°C. For enzymatic deglycosylation, 20 μg of total and 100 μg of biotinylated and avidin precipitated proteins were treated for 1h at 37°C with either endoglycosydase H or PNGase F or control according to the manufacturer’s protocol (New England Biolabs, Ipsxich, MA). For P2Y_14_ antagonist studies, PPTN was dissolved in DMSO and applied to confluent MDCK-C11 cells at a final concentration of 10 μM (0.05% DMSO). Pretreatment with PPTN or vehicle (0.05% DMSO) was for 30 minutes prior to control or UDP-glucose treatment.

### Radioligand binding assays

[^3^H]UDP-glucose binding assays were performed in MDCK-C11 cell line and FACS isolated IC membrane preparations, as previously described [18]. Confluent MDCK-C11 cells were scrapped in ice-cold PBS, pelleted by centrifugation (500 g, 10 min) and then resuspended in 1 ml ice-cold Tris-acetate 0.2 M buffer (pH 7.5) containing protease inhibitors (Complete Mini, Roche, Indianapolis, IN) using a 25G needle. Cell membranes were harvested by passing through a cell cracker (HGM lab equipment, Heidelberg, Germany) 10 times. The solution was then centrifuged 10 min at 17000 *g* and membrane pellets were frozen in liquid nitrogen and kept at −80°C until use. Protein concentration was determined using a nanodrop 2000 (Thermo scientific).

For dose-displacement assays 15 μg of MDCK-C11 cell membrane proteins were incubated for 3 hours at 22°C in a medium containing 50 mM Tris/HCl pH 7.4, 1 mM EDTA, 5 mM MgCl2 and BSA (5 mg/ml), [^3^H]-UDP-glucose (3 nM) and selected concentrations of UDP-glucose or ATP. Incubation was terminated by the addition of ice-cold 50 mM Tris/HCl pH 7.4, 1 mM EDTA, 5 mM MgCl_2_ and was followed immediately by filtration under vacuum through Gelman A/E glass filters (Pall life science, An Arbor, MI) pre-soaked in binding buffer. The filters were rinsed twice before the addition of 5 ml of scintillation fluid (OpticFluor, Groninge, The Netherlands). Receptor-bound radioactivity was measured using liquid scintillation analyzer Tricarb 2200 CA from Parckard. All assays were performed in triplicate. [^3^H]UDP-glucose binding assays were also performed in isolated EGFP(+) and EGFP(−) cells. Membranes were incubated with a saturating concentration of [^3^H]UDP-glucose for 3 hours at 22°C. The non-specific [^3^H]UDP-glucose binding was determined in the presence of 10 μM unlabeled UDP-glucose. The specificity of [^3^H]UDP-glucose binding was demonstrated in the presence of a saturating concentration of ATP (10 μM). Incubations were stopped by the addition of ice-cold buffer and receptor-bound radioactivity was determined as described above. The equilibrium dissociation constant (Kd) and the capacity of binding in dose-displacement studies were calculated using a scatchard plot and are expressed as the mean SD. Statistical analysis were performed using the unpaired Student t-test.

### Immunoblotting

Proteins were run on NuPAGE Novex bis/tris 4–12% gels (Invitrogen) and transferred to nitrocellulose membranes (Bio-Rad). After blocking (5% BSA in TBS 0.1% Tween 20 for 1 h), membranes were incubated overnight with the primary antibody. After 3 washes in TBS 0.1% Tween 20, horseradish peroxidase-conjugated secondary antibodies diluted 1:10,000 in TBS 0.1% Tween 20 were applied for 1 h at RT. Membranes were assayed with Western Lightning Chemiluminescence reagent (Perkin Elmer Life Sciences, Waltham, MA, USA) and Kodak imaging films.

### Immunofluorescence

Mice were anesthetized using pentobarbital sodium (50 mg/kg body, ip). The left kidney was perfused through the renal artery with PBS (0.9% NaCl in 10 mM phosphate buffer, pH 7.4), followed by paraformaldehyde-lysine-periodate fixative (PLP; 4% paraformaldehyde, 75 mM lysine-HCl, 10 mM sodium periodate, and 0.15 M sucrose, in 37.5 mM sodium phosphate) for 10 min at a constant rate of 3.5 ml/min. Kidneys were further fixed by immersion in PLP for 4 h at room temperature and subsequently overnight at 4°C. After extensive washes in PBS, cryo-protection was performed in PBS containing 0.9 M (30% wt/vol) sucrose overnight at 4°C. Prior to cryo-sectioning, tissues were embedded in Tissue-Tek OCT compound 4583 (Sakura Finetek USA, Torrance, CA) and frozen at −20°C. Sections (4–10 μm) were cut on a Leica CM3050-S cryostat (Leica Microsystems, Bannockburn, IL) and stored at 4°C until use [38,44]. Sections were rehydrated in PBS and antigen retrieval techniques were performed by microwave heating in alkaline solution (10 mM Tris buffer, 1 mM EDTA, pH 9.0) 3 times for 1 min, with 5 min interval and then cooled down to room temperature. Sections were then treated with 1% (wt/vol) SDS for 4 min [45]. After washes in PBS, and incubation for 20 min in 1% (wt/vol) BSA in PBS the sections were incubated for 60 min or overnight at 4°C with the primary antibody diluted in PBS containing 1% BSA. The secondary antibody was applied for 1 h at room temperature and slides were mounted in Vectashield H1200 medium containing 4,6-diamidino-2-phenylindole (DAPI) (Vector Laboratories, Burlingame, CA). Digital images were acquired using a Nikon 90i epifluorescence microscope (Nikon Instruments, Melville, NY). Images were analyzed using Volocity version 6.2.1 image-processing software (Perkin Elmer), and imported into Adobe Photoshop software as TIFF files and the levels command was applied to the entire field of view to better represent the raw data visualized under the microscope.

MDCK-C11 cells grown to confluence on filter (Corning) were biotinylated as described above and were then fixed for 30 min in 4% paraformaldehyde (Electron Microscopy Sciences, Hatfield, PA). Cells were washed three times with PBS and treated with 1% SDS for 4 min for antigen retrieval. After several washes in PBS and blocking of the proteins with 1% BSA for 30 min, cells were incubated for 1 h with anti-P2Y_14_ antibody diluted 1:200 in PBS containing 1% BSA. Donkey anti-rabbit Cy3-conjugated antibody (1:800) and FITC-conjugated streptavidin (1:1000) were applied for 40 min at RT. After three washes with PBS, cells were mounted with Vectashield (Vector Laboratories) and visualized with a Zeiss Radiance 2000 laser scanning confocal microscope (Zeiss Laboratories) using LaserSharp 2000 version 4.1 software. Z-series (0.25 µm interval) were taken for X-Z side view representations.

### Statistical analysis

The effects of treatments between two groups were determined by unpaired Student's *t*-test when appropriated. Comparisons between multigroups were determined by one-way ANOVA followed by a post-hoc t-test. All tests were two-tailed, and *P* < 0.05 was considered as statistically significant.

## Results

### P2 receptor mRNA expression in intercalated cells

**Fig 1:**
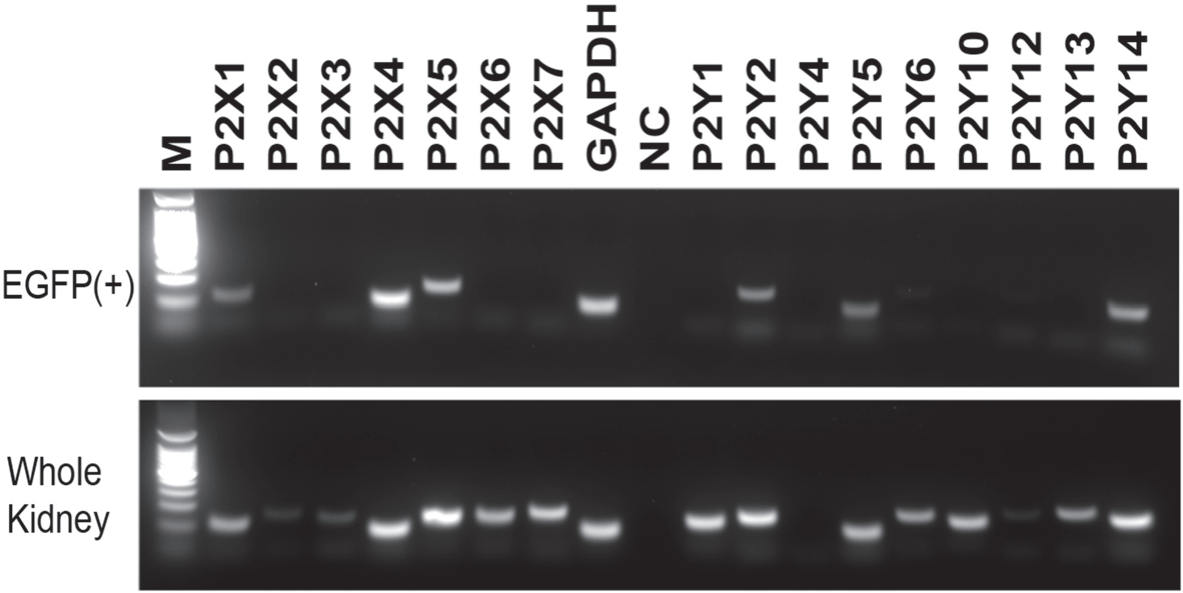
RT-PCR detection of P2 receptors. Lower panel shows RT-PCR in whole kidney and upper panel shows RT-PCR in EGFP(+) cells isolated by FACS from B1-EGFP mouse kidneys. GAPDH was used as a positive control. NC = no template control.

We first analyzed P2X and P2Y receptor mRNA expression by conventional RT-PCR in IC-enriched EGFP(+) cells isolated by FACS from the kidneys of B1-EGFP mice as well as in whole kidney. In these mice, EGFP expression is driven by the promoter of the V-ATPase B1 subunit, and occurs specifically in type A intercalated cells (A-ICs), type B intercalated cells (B-ICs), and in the connecting tubules (CNT) [41]. We have previously shown that we can generate a highly enriched EGFP(+) cell preparation that is depleted of all other cell types after FACS isolation [42,44]. As shown in Fig. 1, while transcripts specific for all P2 receptors tested were detected, except P2Y_4_, in whole kidney extracts (bottom panel), this number was narrowed down to 3 P2X (P2X_1_, P2X_4_ and P2X_5_) and 3 P2Y (P2Y_2_, p2y5 and P2Y_14_) receptors in EGFP(+) cells (Fig. 1, top panel). The full sequence of murine P2Y_11_ has not been fully identified when we initiated the present work, and future studies will be required to characterize its expression in ICs.

**Fig 2:**
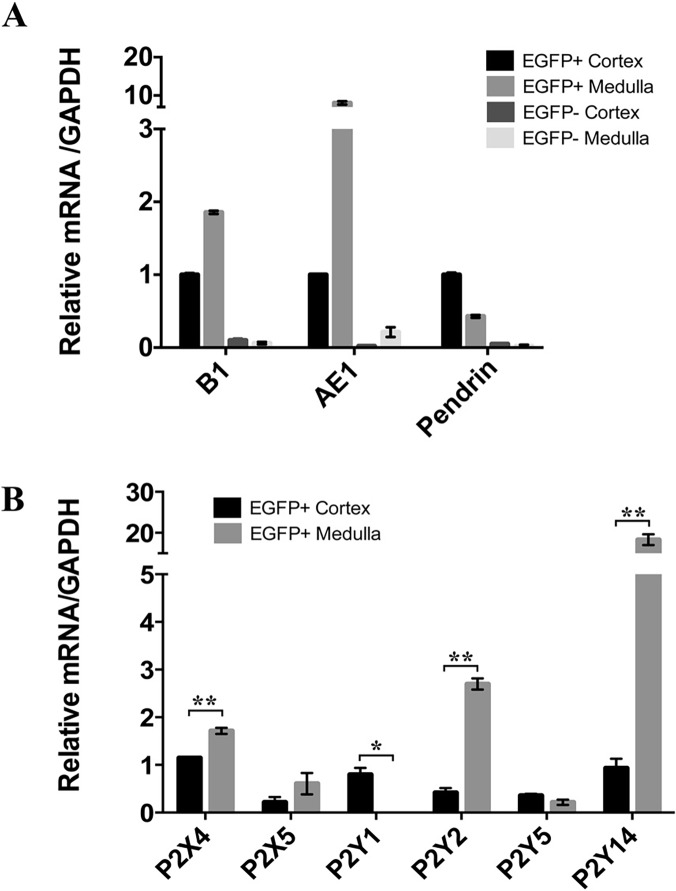
Quantitative PCR analysis of EGFP(+) cells isolated from the kidney cortex and medulla. (A) Detection of IC markers (B1, AE1, pendrin) in EGFP(+) vs EGFP(−) cells isolated from renal cortex and medulla. Values are normalized to GAPDH and represented as fold changes relative to the values obtained in cortical EGFP(+) cells. (B) Relative P2 receptor mRNA levels, analyzed by quantitative PCR in medullary and cortical EGFP(+) cells. Data are normalized for GAPDH and are expressed as mean ± SEM (n = 3), *P<0.05, **P< 0.001.

We then took advantage of the regional separation of B-ICs and CNT cells, which are located exclusively in the renal cortex and to a lesser extent in the outer stripe of the outer medulla (OS), and A-ICs, which are located in all kidney regions except the tip of the papilla [46,47]. A mixed EGFP(+) cell population containing A-ICs, B-ICs and CNT cells was isolated from the kidney cortex and was compared with an A-IC enriched population isolated from the inner stripe of the outer medulla (IS) and inner medulla. Quantitative PCR showed a marked enrichment of mRNA transcripts specific for the V-ATPase B1 subunit (a marker of A-ICs, B-ICs and CNT cells) [48–50], AE1 (a marker of A-ICs) [51] and pendrin (a marker of B-ICs) [52,53] in EGFP(+) cells compared to EGFP(−) cells isolated from the same regions (Fig. 2A). As expected, an increase in AE1 mRNA and a decrease in pendrin mRNA were detected in EGFP(+) cells isolated from the medulla versus the cortex, respectively, demonstrating the enrichment of A-ICs in the medullary EGFP(+) cells versus cortical EGFP(+) cell populations. Quantitative PCR showed significantly higher expression levels for P2X_4_, P2Y_2_ and P2Y_14_ in EGFP(+) cells isolated from the medulla compared to the cortex (Fig. 2B). P2X_5_ and p2y5 expression appeared to be similar in both regions and P2Y_1_ was detectable only in cortical EGFP(+) cells, suggesting that A-ICs most probably do not express P2Y_1_. The very high P2Y_14_ enrichment (by more than 20 fold) that we measured in medullary EGFP(+) cells compared to cortical EGFP(+) cells prompted us to further characterize the role of this receptor in ICs.

### P2Y14 is exclusively expressed in intercalated cells

**Fig 3:**
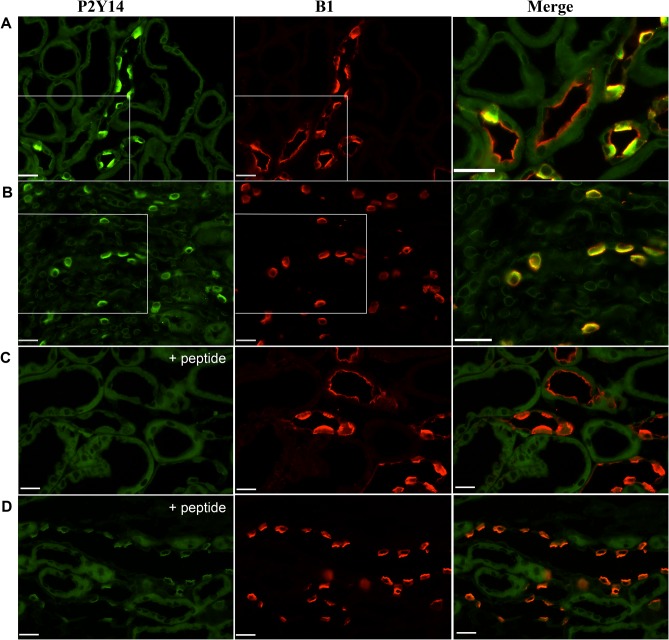
Immunofluorescence localization of P2Y_14_ in mouse kidney. Cortical (A) and medullary (B) sections double-labeled for P2Y_14_ (green) and the V-ATPase B1 subunit (red). P2Y_14_ was detected in ICs identified by their positive labeling for the V-ATPase (yellow in the merge panels shown in A and B). No P2Y_14_ was detected in distal tubule cells, which also express the V-ATPase (red in the merge panel shown in A). The P2Y_14_ staining was abolished after pre-incubation of the P2Y_14_ antibody with its immunizing peptide in the cortex (C) and medulla (D). Scale bars = 25 μm.

**Fig 4:**
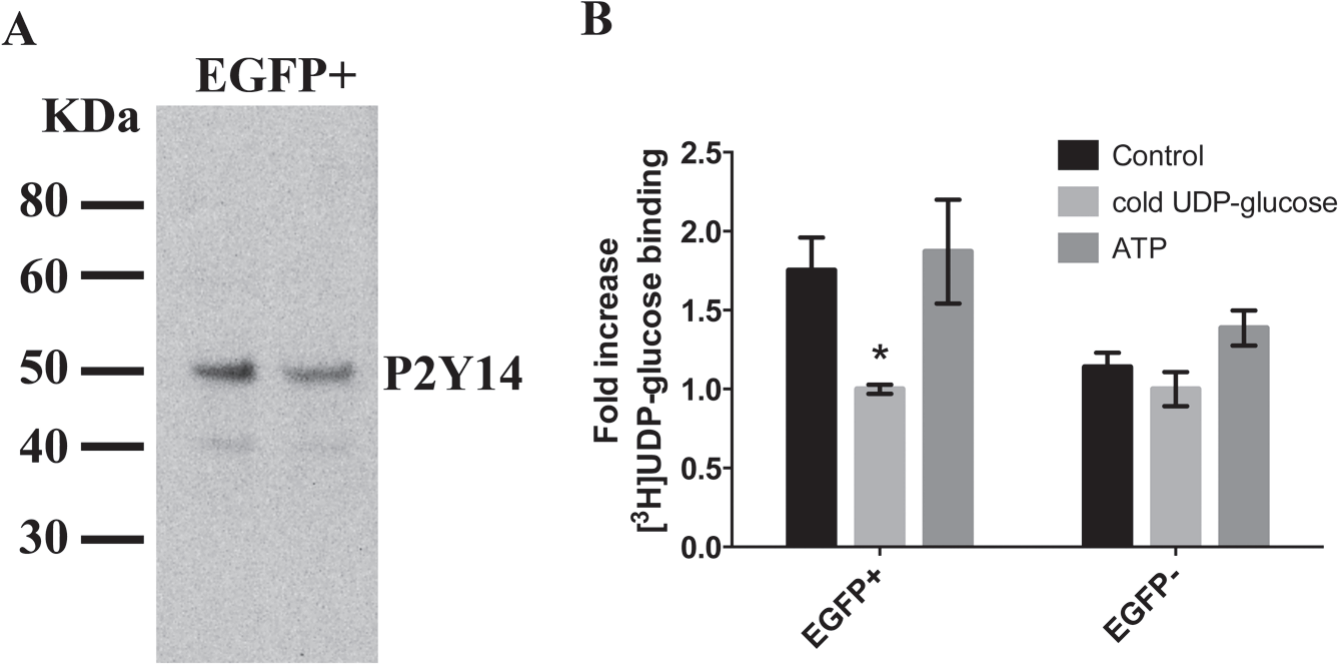
Expression of P2Y_14_ in EGFP(+) cells. (A) Representative immunoblot profile of P2Y_14_ in two EGFP(+) cell samples isolated by FACS. (B) Binding of [^3^H]UDP-glucose to total membranes prepared from FACS isolated EGFP(+) and EGFP(−) cells in the presence or absence of a saturating concentration (10^–5^ M) of unlabeled UDP-glucose or ATP. Data are represented as fold changes compared to the binding measured in the presence of unlabeled UDP-glucose. Each bar represent the average of 3 independent experiments each performed in triplicate. Values are expressed as mean ± SEM, * P<0.05.

Double-immunofluorescence labeling of kidney sections for P2Y_14_ (green) and the V-ATPase B1 subunit (red) showed specific expression of P2Y_14_ in A-ICs and B-ICs in the cortex, while no P2Y_14_ was detected in the distal and connecting tubules (Fig. 3A). In the medulla, P2Y_14_ was detected only in A-ICs (Fig. 3B). P2Y_14_ was expressed in the apical region of both A-ICs and B-ICs, as compared to the V-ATPase B1 subunit, which is apical in A-ICs, but basolateral or bi-polar in B-ICs [48]. Pre-incubating the P2Y_14_ antibody with its immunizing peptide abolished the P2Y_14_ staining in both the cortex (Fig. 3C) and medulla (Fig. 3D). P2Y_14_ was also detected in occasional immune cells, which remained attached to the blood vessel walls (data not shown), consistent with its previously described localization in circulating immune cells [30–32]. Immunoblotting of EGFP(+) cell extracts with the P2Y_14_ antibody showed a predominant 50-KDa band and a weaker 40-kDa band (Fig. 4A). We then performed a radiolabeled UDP-glucose binding assay using total membranes separated from FACS isolated EGFP(+) cells and EGFP(−) cells (Fig. 4B). The [^3^H]UDP-glucose binding measured in EGFP(+) cells was displaced with a saturating concentration of cold UDP-glucose [10^–5^ M], but not with ATP [10^–5^ M], showing UDP-glucose-specific binding. In contrast, in EGFP(−) cells no UDP-glucose-specific binding was measured. Altogether, these data show that ICs are the only renal epithelial cells that express P2Y_14_.

### **P2Y**_14_ **activation up-regulates pro-inflammatory chemokine mRNAs in intercalated cells**

**Fig 5:**
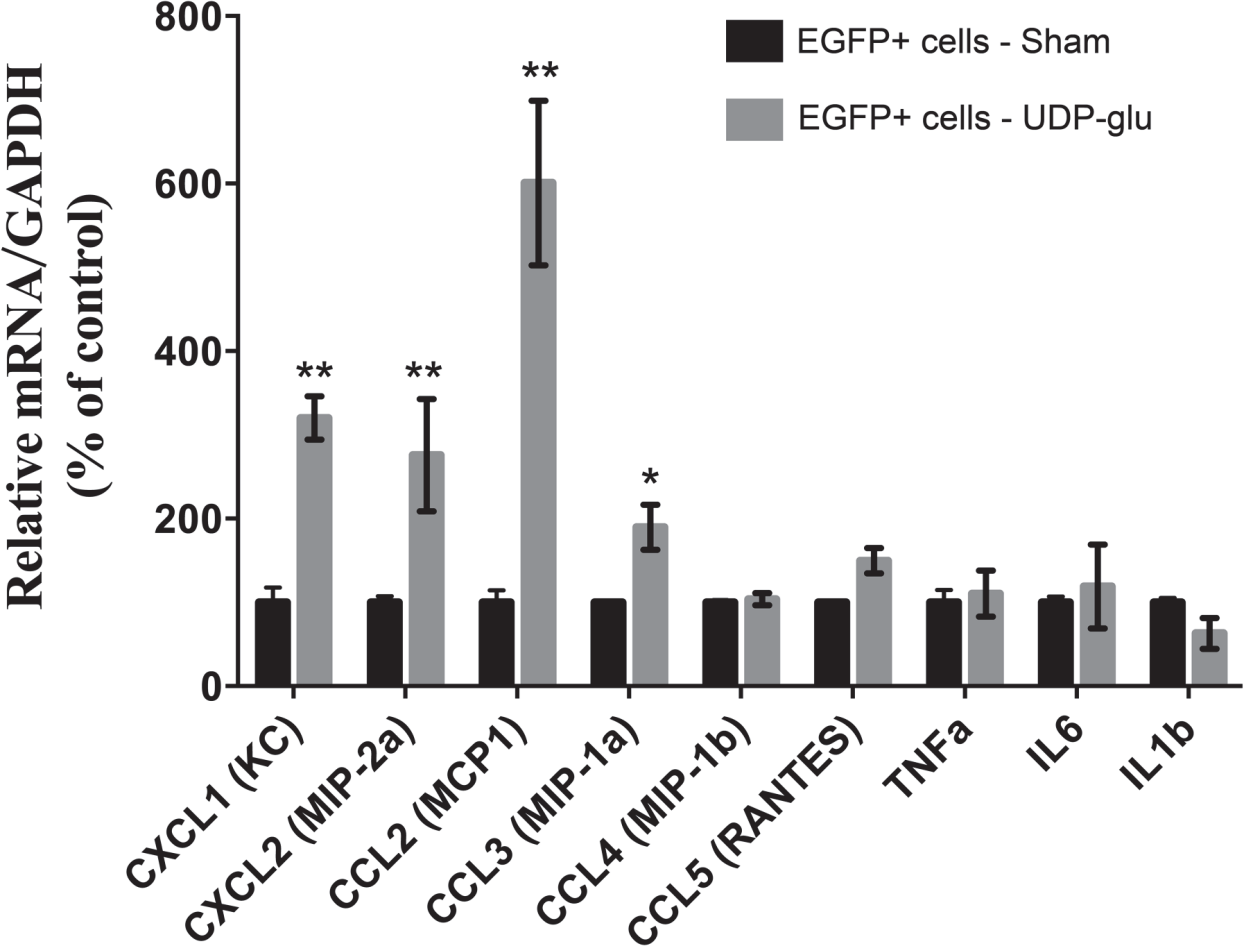
Quantitative PCR detection of pro-inflammatory mediators in EGFP(+) cells. EGFP(+) cells were isolated by FACS from B1-EGFP mice 4h after an i.v. injection with saline (sham) or with saline containing 100 μM UDP-glucose (UDP-glu). All values are normalized to GAPDH. Data are represented as % changes relative to control. Values are mean ± SEM (n = 4), *P<0.05, ** P<0.001.

To determine whether P2Y_14_ activation induces the upregulation of pro-inflammatory mRNAs in ICs *in vivo*, we injected B1-EGFP mice through the tail vein with either a saline solution (sham) or a solution containing 100 μM UDP-glucose. Kidneys were harvested 4 h later and processed for FACS isolation of EGFP(+) cells, mRNA extraction and real-time PCR to measure pro-inflammatory chemokine and cytokine expression. To avoid contamination of blood immune cells, which also express P2Y_14_, we flushed the blood out of the kidneys by perfusing mice with PBS through the cardiac left ventricle. In addition, maximum purity (>95%) of EGFP(+) cells was obtained by restricting the sorting parameters to isolate only the brightest EGFP(+) cells. Cytospin smears of EGFP(+) cells immunostained for CD45 (a marker of leukocytes) did not show any contamination of the samples with leukocytes (data not shown). We were thus confident that any changes in pro-inflammatory mediator expression following P2Y14 activation with UDP-glucose were attributed to the presence of the receptor in ICs uniquely. As shown in Fig. 5, the neutrophil chemo-attractants, CXCL1 (KC) and CXCL2 (MIP-2α) (both murine homologues of IL-8) had significantly higher expression levels following UDP-glucose treatment in vivo for 4 hours. A significant increase was also observed for the monocyte chemo-attractant CCL2 (MCP-1) and CCL3 (MIP-1α). No effect was observed after 2, 6, or 12 hours (data not shown). In addition, no significant changes were observed for CCL4, CCL5, TNFα, IL1β, and IL6 expression at any time point. These results show that ICs produce pro-inflammatory mediators *in vivo* following activation by a pro-inflammatory agonist, and that this process can be efficiently promoted through UDP-glucose/P2Y_14_ signaling.

### **Characterization of the P2Y**_14_ **signaling pathway in the renal epithelial cell line MDCK-C11**

**Fig 6:**
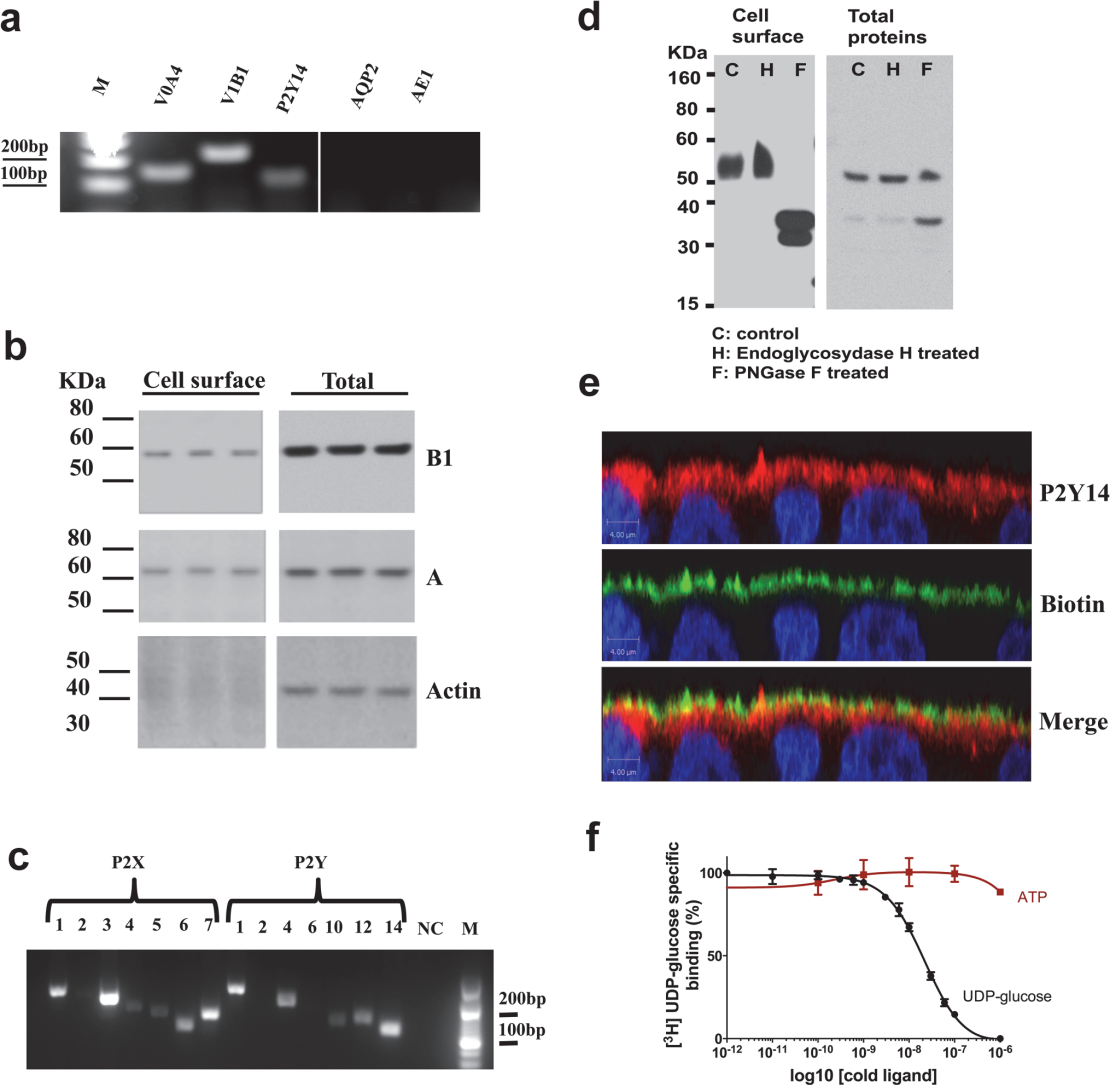
P2Y_14_ expression in MDCK-C11 cells. (a) RT-PCR analysis of IC markers including the V-ATPase a4 subunit (V0A4), the V-ATPase B1 subunit (V1B1) and AE1, and the principal cell marker aquaporin 2 (AQP2), as well as P2Y_14_ in MDCK-C11 cells. (b) Representative immunoblots following plasma membrane biotinylation showing cell surface versus total protein expression of the V-ATPase B1 and A subunits, and actin. (c) RT-PCR detection of P2 receptors in MDCK-C11 cells. (d) Immunoblot profile of P2Y_14_ expression in MDCK-C11. Plasma membrane (left) and total cell expression (right) are represented under control conditions (C) and after treatment with endoglycosydase H (H) and PNGase F (F). (e) X-Z confocal microscopy representation of MDCK-C11 cells grown on filter, showing P2Y_14_ expression (red). Plasma membrane is labeled with biotin-streptavidin FITC (green). The merge panel shows partial co-localization of P2Y_14_ with biotin in the apical membrane (orange/yellow) as well as sub-apical localization (red). Scale bars = 4 μm. (f) Concentration-dependent inhibition of [^3^H]UDP-glucose binding to MDCK-C11 membranes by unlabeled ligands. Membranes (15 μg protein) were incubated for 3 hours at 22C with [^3^H]UDP-glucose (3 nM) and increasing concentrations of UDP-glucose or ATP. Each point represents the average of 4 independent experiments performed in triplicate. The data are expressed as values relative to the total binding observed in the absence of unlabeled ligand and are corrected for non specific binding determined in the presence of a saturating concentration of UDP-glucose (10 μM).

We then used the Madin-Darby Canine Kidney (MDCK) subclone C11 (MDCK-C11) as a renal epithelial cell model for the initial identification and characterization of the P2Y_14_ signaling pathway. MDCK-C11 cells were previously shown to possess some characteristics of ICs, including Cl^−^ and H^+^ secretion and the activation of H^+^ secretion by cAMP [54]. In agreement with this notion, RT-PCR analysis showed expression of markers of ICs, including the V-ATPase B1 and a4 subunits in these cells (Fig. 6A). AQP2, a marker of collecting duct principal cells, was not detected, supporting their non-principal cell phenotype [54]. MDCK-C11 cells did not express AE1 and thus do not retain all characteristics of A-ICs. However, expression in MDCK-C11 cells of the V-ATPase B1 and A subunits was confirmed at the protein level by western blotting, in total cell lysates and in a biotinylated plasma membrane protein fraction (Fig. 6B). The same membranes were re-blotted for actin. The absence of actin staining in the plasma membrane preparation showed the absence of biotin contamination of intracellular proteins in this fraction. RT-PCR analysis showed transcripts specific for P2Y_1_, P2Y_4_, p2y10, P2Y_12_ and P2Y_14_ (Fig. 6C). With the exception of P2X_2_ all other P2X receptors were also detected. Western blotting analysis showed expression of P2Y_14_ protein in total cell lysates and at the cell surface (Fig. 6D). In agreement with a previous report in glioma C6 cells [55], we detected a more diffuse band at higher molecular weight in the plasma membrane compared to the 50 kDa band in total cell lysates, indicating that only the glycosylated receptor can reach the cell surface. Glycosylation of P2Y_14_ was confirmed by PNGaseF treatment, which resulted in the appearance of the deglycosylated form of P2Y_14_ at around 45KDa in total cell lysates and in cell surface protein extracts. Confocal microscopy showed P2Y_14_ localization in the apical plasma membrane (red), which was biotinylated and labeled with biotin (green) in MDCK-C11 cells grown on filters (Fig. 6E). P2Y_14_ is also expressed in the sub-apical region of the cells. We then investigated the functionality of the receptor in MDCK-C11 cells by using a radiolabeled UDP-glucose binding assay. The specificity of UDP-glucose binding was analyzed by dose-displacement of [^3^H]UDP-glucose in the presence of unlabeled UDP-glucose or ATP. As shown in Fig. 6F, the addition of increasing concentrations of unlabeled UDP-glucose diminished [^3^H]UDP-glucose binding in a dose-dependent manner. Concentrations of ATP of up to 10 μM had no significant inhibitory effect on the [^3^H]UDP-glucose binding. Scatchard analysis of the binding shows only one class of binding site with an affinity of 11.5 ± 1.3 nM and a maximal binding capacity of 16.0 0.2 pmol/mg of protein. These values are very similar to the values obtained in HEK-293 cells expressing P2Y_14_ (previously known as KIAA0001) [18].

### **UDP-glucose increases ERK1/2 phosphorylation through P2Y**_14_ **activation in MDCK-C11 cells**

**Fig 7:**
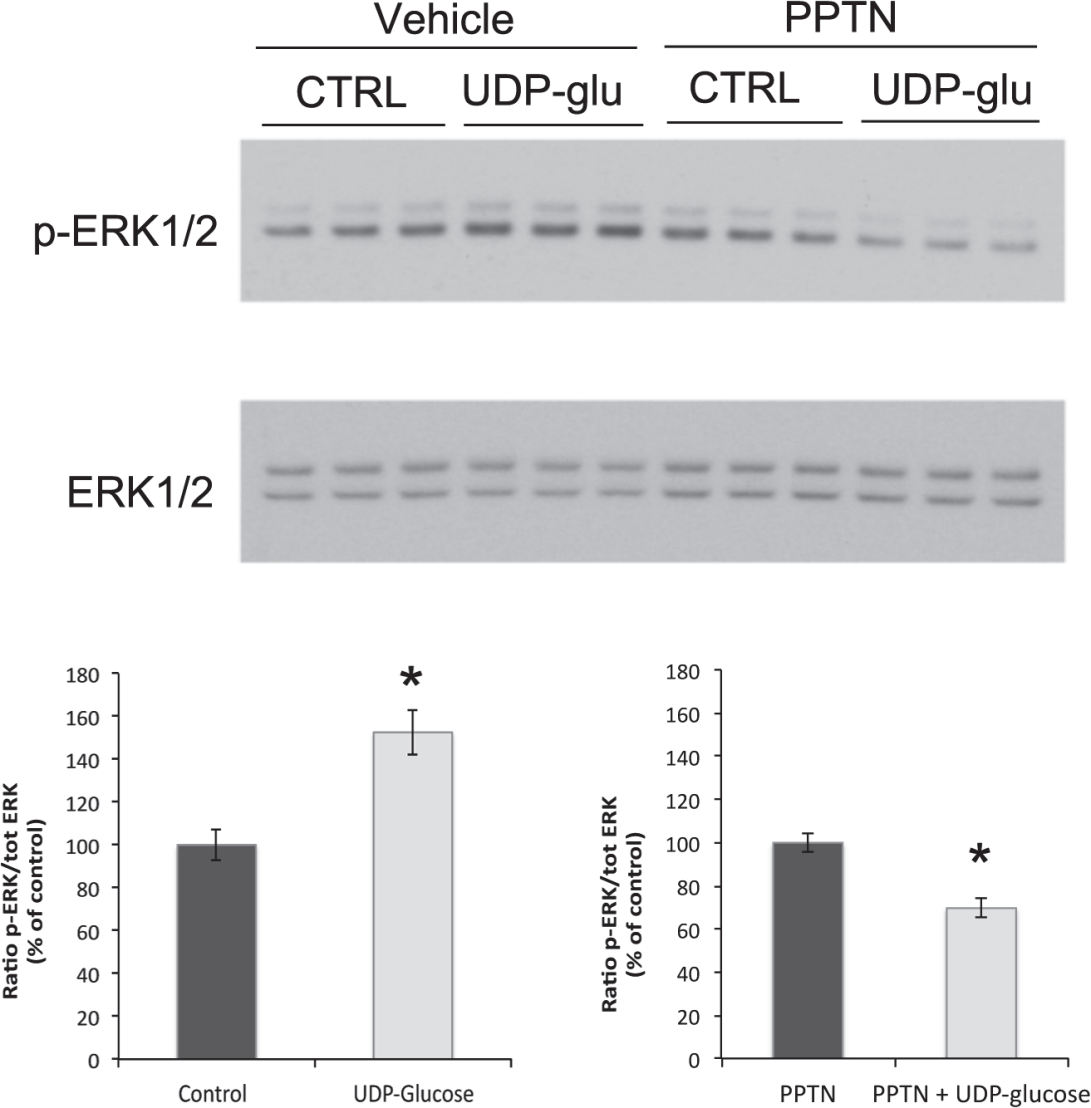
P2Y_14_ activation by UDP-glucose increases ERK1/2-phosphorylation in MDCK-C11 cells. Representative immunoblots showing triplicates of ERK1/2 phosphorylation (upper lane) versus total ERK1/2 (lower lane) in cells pretreated with vehicle or the P2Y_14_ antagonist PPTN (10 μM), in the absence (CTRL) or presence of 100 μM UDP-glucose (UDP-glu). Quantification of the ratio of p-ERK/total ERK showed that UDP-glucose induced a significant increase in ERK1/2 phosphorylation (lower left panel, n = 7) and that PPTN prevented the increase in ERK1/2 phosphorylation induced by UDP-glucose (lower right panel, n = 5). Values are represented, relative to either control or PPTN alone, as means ± SEM, * p < 0.005.

As shown in Fig. 7 (upper panel), UDP-glucose (100 μM) induced a significant increase in ERK1/2 phosphorylation in MDCK-C11 cells. This was prevented by pre-treatment with the P2Y_14_ antagonist, 4-((piperidin-4-yl)-phenyl)-(7-(4-(trifluoromethyl)-phenyl)-2-naphthoic acid (PPTN, 10 μM). Quantification of the ratio of p-ERK1/2 to total ERK1/2 showed a significant increase in ERK1/2 phosphorylation following UDP-glucose treatment, which was abolished in the presence of PPTN (Fig. 7, bottom panels). We did not detect an increase in the phosphorylation of other MAPK targets such as p38 and JNK/SAPK (data not shown), consistent with a previous report [56]. In addition, we found no effect of UDP-glucose on IkBα protein expression in MDCK-C11 cells (not shown) suggesting that activation of P2Y_14_ does not stimulate the NF-kB pathway.

### **P2Y**_14_ **activation up-regulates pro-inflammatory chemokine mRNAs through ERK-phosphorylation in MDCK-C11 cells**

**Fig 8:**
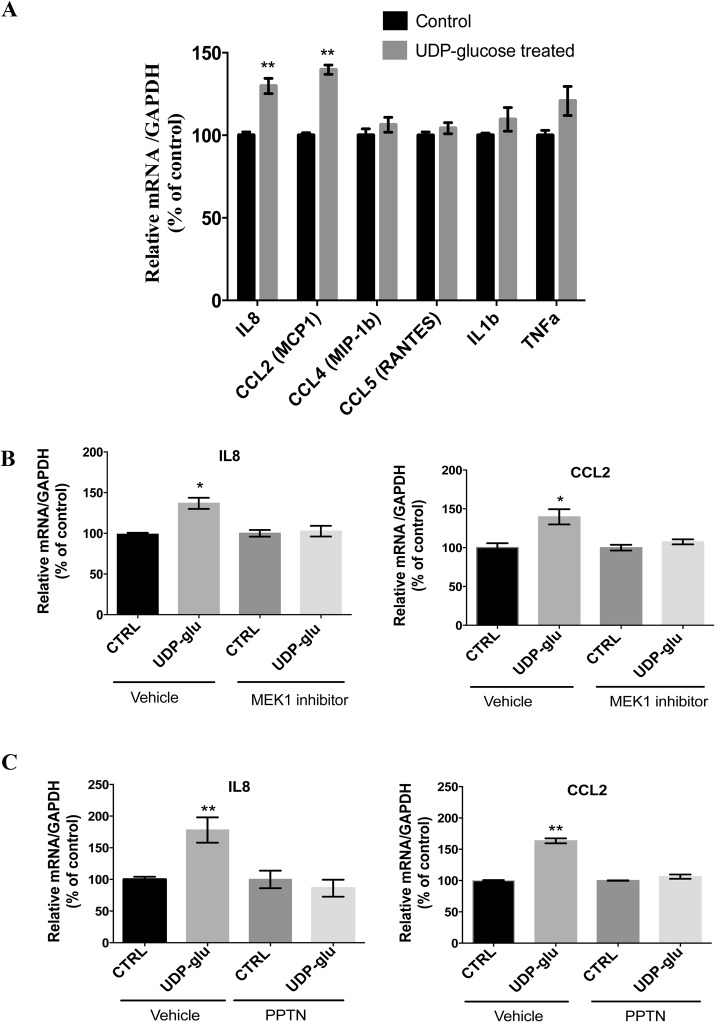
Quantitative PCR detection of pro-inflammatory mediators in MDCK-C11 cells. (A) Detection of mRNA transcripts specific for IL-8, CCL2, CCL4, CCL5, IL1b and TNFa under control conditions and 4h after 100 μM UDP-glucose treatment. Data are represented as % changes relative to control. All values are normalized to GAPDH and are shown as Means ± SEM (n = 5), ** P<0.001. (B) Quantification of changes in IL-8 (left) and CCL2 (right) mRNA expression in MDCK-C11 cells pretreated with the vehicle only or with the MEK inhibitor, PD98059 (50 μM) for 30 minutes in the absence (CTRL) or presence of 100 μM UDP-glucose (UDP-glu). (C) Quantification of changes in IL-8 (**left**) and CCL2 (**right**) mRNA expression in MDCK-C11 cells pretreated with the vehicle only or with PPTN (10 μM) for 30 minutes, in the absence (CTRL) or presence of 100 μM UDP-glucose (UDP-glu). Data are represented as % changes relative to control. Values are means ± SEM (n = 3), * P<0.05, **P<0.001.

Several studies have suggested a role for P2Y_14_ as a mediator of inflammation [34,35,37,57]. We assessed here the effects of P2Y_14_ activation with UDP-glucose on mRNA expression of several pro-inflammatory chemokines and cytokines in MDCK-C11 cells (Fig. 8). As shown in Fig. 8A, MDCK-C11 cells increased IL-8 and CCL-2 mRNA expression following 4 h treatment with 100 μM UDP-glucose. IL-8 and CCL-2 (also known as MCP-1) are well-known chemo-attractants for neutrophils and monocytes, respectively. There was no detectable increase of other pro-inflammatory mediators such as CCL4, CCL5, IL1β or TNFα. We then determined the contribution of ERK-phosphorylation in cytokine production by using the MEK inhibitor PD98059 [58]. As shown in Fig. 8B, PD98059 abolished the UDP-glucose induced up-regulation of IL-8 and CCL-2. Pretreatment of MDCK-C11 cells with the P2Y_14_ antagonist PPTN [37] also abolished the increase in IL8 and CCl2 mRNA expression induced by UDP-glucose (Fig. 8C).

### UDP-glucose activation induces neutrophil infiltration into the kidney medulla

**Fig 9:**
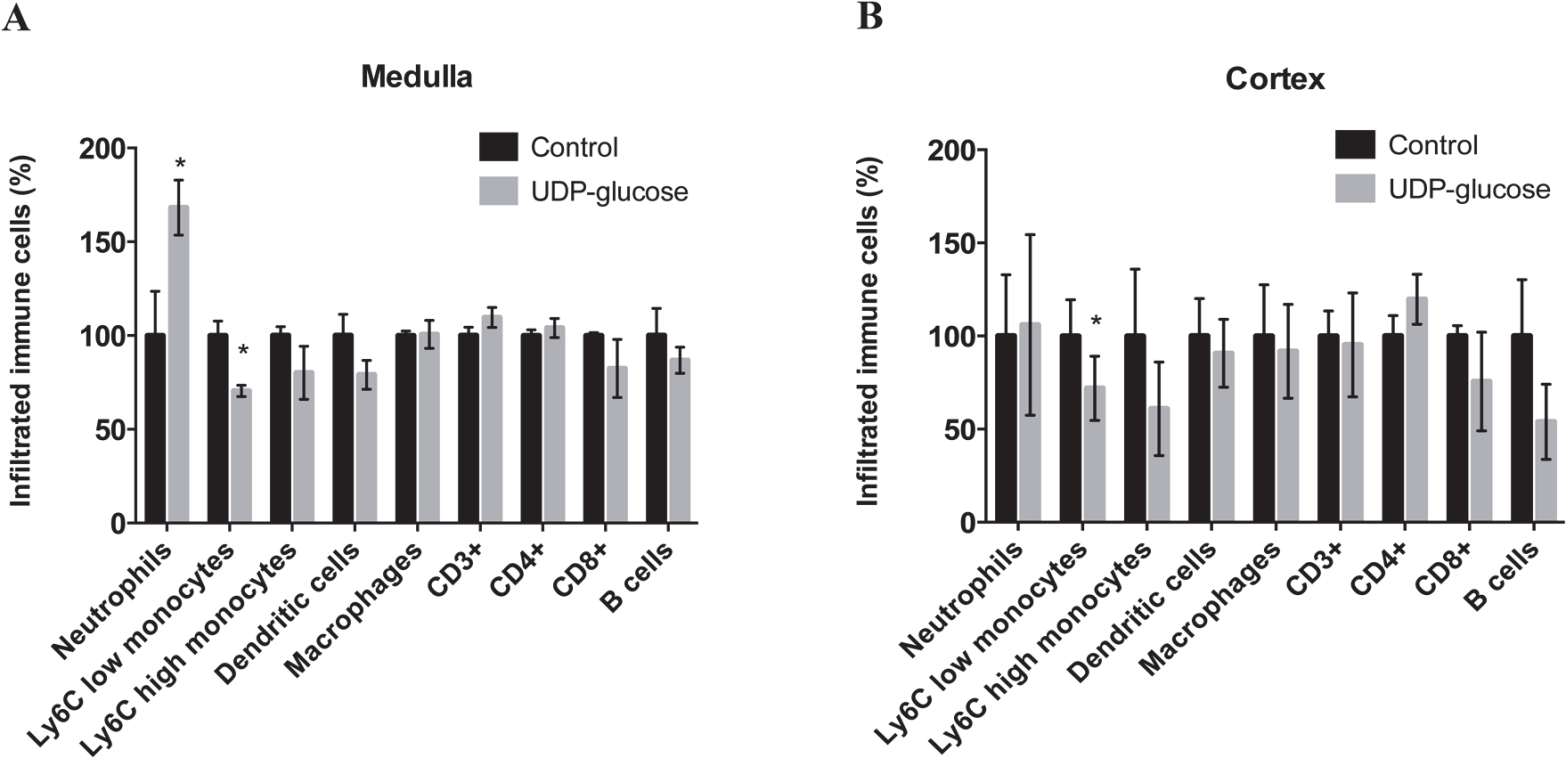
Flow cytometry analysis of immune cell infiltration in the kidneys of mice 48 h after injection with saline (sham) or 100 μM UDP-glucose (UDP-glu). Changes in kidney medulla (A) or cortex (B) infiltrated immune cell counts are represented as % changes relative to control. Values are means of percent of each cell population ± SEM from 4–6 animals. *P<0.05.

The pathophysiological relevance of P2Y_14_ expression in ICs was assessed by measuring the infiltration of immune cells into the kidney using flow cytometry in mice that were challenged with an injection of UDP-glucose. To address the spatial distribution of immune cells in the kidney, we separated the kidney into cortex and medulla. B1-EGFP mice were challenged with a tail-vein injection of either 100 μM UDP-glucose (treated) or saline (sham), as discussed above. Mice were perfused 48 h later through the cardiac left ventricle with PBS to flush the blood out of the kidney vessels and hence measure only tissue infiltrated immune cells. This approach identified a significant and selective accumulation of neutrophils in the kidney medulla (Fig. 9A). This observation is consistent with the upregulation of neutrophil chemo-attractants observed in EGFP(+) cells after UDP-glucose activation. Anti-inflammatory (Ly6C low) monocytes also slightly decreased in the medulla, but no other significant changes in other immune cell counts were observed. In the kidney cortex, the numbers of most cell types remained unchanged upon UDP-glucose treatment, except for the Ly6C low monocyte population, which decreased slightly (Fig. 9B). No apparent changes in the number of infiltrated immune cells were observed in the cortex and medulla at earlier time points (12 and 24 hours) following UDP-glucose injection (data not shown).

### The UDP-glucose induced neutrophil infiltration occurs primarily in the renal medulla

**Fig 10:**
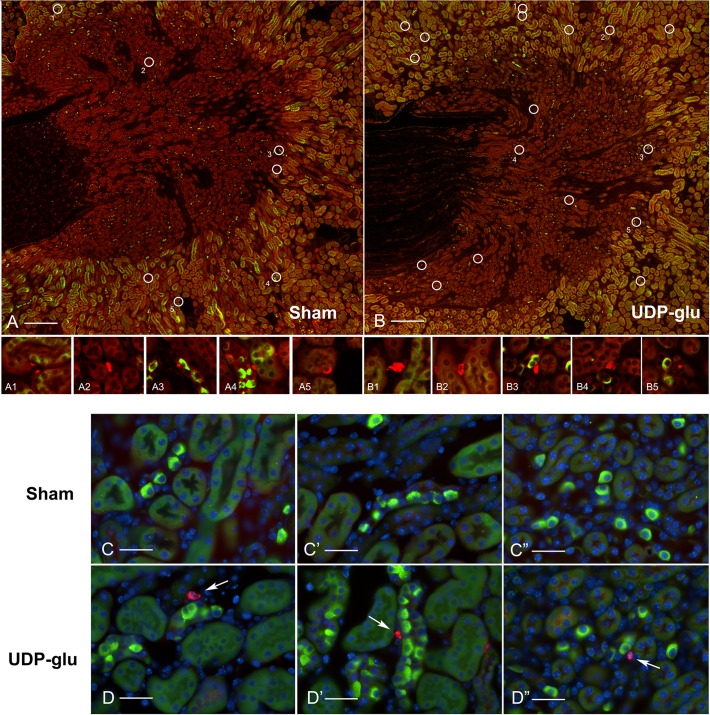
Immunolocalization of neutrophils in kidney medulla of mice 48 h after injection with saline (Sham) or 100 μM UDP-glucose. Mosaic images of kidney medulla double-labeled for P2Y_14_ (green) and the neutrophil marker Ly6G (red; white circles) from mice injected with saline (A) or 100 μM UDP-glucose (B). Individual neutrophils, delineated in the corresponding white circles in Panels A and B, are shown in the small panels A1-A5, and B1-B5, respectively. (C-D) High magnification images showing the presence of neutrophils (arrows) in proximity to medullary ICs after UDP-glucose injection (D, D', D"). In the sham animals (C, C', C"), the areas surrounding collecting ducts were often devoid of neutrophils. Scale bars = 200 μm in A and B, 25 μm in C-D.

Kidney sections from control and UDP-glucose treated mice were stained for P2Y_14_ (green) and for Ly6G, a neutrophil marker (red). Mosaic images were captured and neutrophils identified based on their positive labeling for Ly6G and their poly-nucleated phenotype (white circles) (Fig. 10). More neutrophils were visible in the medulla of UDP-glucose treated mice (Fig. 10B) compared to sham-treated control mice (Fig. 10A). Panels A1-A5 and B1-B5 show the individual neutrophils delineated in the circles shown in Panels A and B, respectively. At higher magnification, neutrophils were sometimes seen in the proximity of intercalated cells in the renal medulla of UDP-glucose treated mice (Figs. 10D, 10D', 10D"), while in control mice, collecting ducts were often seen with no surrounding neutrophils (Fig. 10C, 10C', 10C").

## Discussion

In this study, we characterized the P2 receptor profile of renal intercalated cells, and showed that they express high levels of the pro-inflammatory P2Y_14_ receptor. In addition, our data demonstrate that P2Y_14_ activation by UDP-glucose in ICs induces an inflammatory response mediated by chemokine upregulation and neutrophil recruitment into the kidney medulla. Neutrophil recruitment was demonstrated using two complementary approaches: quantitative flow cytometry analysis, and immunofluorescence microscopic visualization of Ly6G-positive neutrophils on kidney sections. Our study, therefore, identifies ICs as potential sensors, mediators and effectors of sterile inflammation in the kidney. Our data suggest that the contribution of this novel pathway should now be examined in different models of kidney disease.

While we detected numerous P2 receptors in mRNA samples isolated from the entire kidney, we found only 6 (P2X_1_, P2X_4_, P2X_5_, P2Y_2_, p2y5 and P2Y_14_) receptors in ICs. The expression of P2Y_2_ in these cells is in agreement with a previous pharmacological study showing functional P2Y_2_ in the apical membrane of ICs in rabbit CCDs [59]. P2Y_2_ is traditionally viewed as a regulator of collecting duct principal cells, but our data further support its participation in IC function. Activation of this receptor by UTP, ATP and guanosine activates the ERK1-2/MAPK pathway in astrocytes [60], and it would be interesting to determine whether P2Y_2_ plays a similar role in ICs. Interestingly, while P2X_7_ and P2X_6_ have been described in collecting ducts [61,62], we did not detect these receptors in EGFP(+) cells, illustrating the importance of conducting cell-specific gene expression analysis. Among the receptors that were detected in ICs, P2X_4_ is regulated by extracellular pH [63] and it will be interesting to determine its role in the acidifying function of ICs. We also observed several purinergic receptors, including P2Y_14_, in MDCK-C11 cells, making them suitable models to characterize this receptor *in vitro*. Nonetheless, the limitations of cell culture models in general must be considered when comparing *in vitro* studies with the *in vivo* situation. The presence of other purinergic receptors, either in ICs or in MDCK-C11 cells, should not affect the outcome of the present study, because the agonist used here, UDP-glucose, was shown to activate P2Y_14_ exclusively.

Infection and ischemic kidney injury stimulate a potent inflammatory response including a rapid infiltration of neutrophils into the affected tissue [64]. Ascending pathogens induce renal epithelial cell damage [65–67]. The role of ICs in the defense against these pathogens has recently emerged by the discovery that they express the antimicrobial agent RNAse 7 and its modulator, the ribonuclease inhibitor (RI) [68–70]. It was proposed that ICs are involved in the maintenance of luminal sterility in the kidney. Uropathogenic *Escherichia coli* can adhere to the apical surface of medullary ICs, where it activates TLR4-dependent and independent signaling pathways [71]. In a more recent study, ICs were shown to limit renal bacterial infection via TLR4 activation, followed by stimulation of the NF-kB pathway and release of the bacteriostatic LCN2 (also known as NGAL) and protons into the lumen [72]. Multiple other studies have characterized Toll-like receptors (TLR) as pathogen recognition and inflammation mediators in the kidney [71,73,74]. However, the molecular mechanisms that stimulate inflammation secondary to "pathogen-free" kidney diseases, nephrotoxicity and renal transplantation among others are poorly understood. Our present study provides evidence for a parallel non-TLR mediated inflammatory pathway determined by ICs. We suggest that the UDP-glucose released from damaged cells activates P2Y_14_ in ICs to initiate an inflammatory response via the production of pro-inflammatory chemokines, which then recruit neutrophils to damaged and/or infected areas. We did not detect P2Y_14_ in neutrophils, so it is unlikely that UDP-glucose acted directly on these cells to induce chemotaxis in an autocrine manner. We, therefore, propose that P2Y_14_ receptors act as danger sensors in kidney ICs. Intriguingly we found that P2Y_14_ mRNA expression is 20 times higher in medullary ICs compared to cortical ICs, despite the fact that immunostaining detected abundant protein in both cortical and medullary ICs. This could reflect a higher level of mRNA expression in A-type ICs, which are enriched in the isolated medullary preparation compared to the cortical EGFP(+) cells. Alternatively, the renal medulla is the primary site of exposure to urinary ascending pathogens, which induce renal epithelial cell damage [65–67]. The large amount of P2Y_14_ mRNA seen in this region could be explained by the necessity to synthetize a functional protein immediately following infection, in order to induce a rapid inflammatory response. It will be interesting to test the possibility that the ascending bacteria themselves might release UDP-glucose into the luminal compartment, thereby initiating an inflammatory response in ICs secondary to infections.

P2Y_14_ activates the MAPK pathway, which regulates the stability of IL-8 mRNA as opposed to LPS, which transcriptionally activates the IL-8 gene via the NF-kB pathway [75]. In agreement with this notion, we show here that UDP-glucose administered both in vitro and in vivo induces a potent inflammatory response in ICs by up-regulating pro-inflammatory chemokine expression through MAPK activation and subsequent recruitment of neutrophils into the renal medulla. This would suggest that P2Y_14_ (MAPK activation) and LPS (mainly NF-kB activation) act in parallel to increase IL-8 secretion and neutrophil recruitment. Alternatively, P2Y_14_ receptor activation was previously shown to reduce adenylyl cyclase activity leading to reduction of intracellular cAMP [24] and increase of intracellular calcium [34]. Future studies will be required to determine the role of the cAMP/PKA pathway in mediating some of the inflammatory effects observed here. We have previously shown that cAMP induces the apical accumulation of V-ATPase in type A ICs [76]. It will be interesting to determine whether a reduction in cAMP in response to P2Y_14_ receptor activation would "switch" the phenotype of ICs from being proton secreting cells to pro-inflammatory mediators.

Cytokines are important contributors to the initiation of inflammation in the injured kidney. Pro-inflammatory cytokines such as TNFα, IL6 and IL1β induce chemokines through complementary activation, and the NF-kB and TLR related pathways. In our study we show that the increase in neutrophil and monocyte chemo-attractants is not accompanied by an increase of IL1β, TNFα or IL6 expression in ICs, indicating that UDP-glucose can itself act as a pro-inflammatory mediator bypassing the cytokine effects. We suggest that by their ability to produce chemokines, ICs act as immune defense cells by creating a chemotaxic gradient favorable to neutrophil recruitment.

The increase in the amount of infiltrated neutrophils that we observed in the kidney medulla following UDP-glucose administration is in quantitative agreement with previous studies showing a 3-fold increase in renal neutrophil content after bilateral ischemia-reperfusion [77], and a 4-fold increase in the mouse uterus after UDP-glucose administration [35]. In addition, we found significant neutrophil infiltration after 48 hours, a time course identical to the infiltration observed in the mouse uterus [35].

In conclusion, our data suggest that ICs are DAMP sensors that mediate the recruitment of pro-inflammatory neutrophils to the kidney via activation of P2Y_14_. Almost all kidney diseases trigger a strong inflammatory response that can ultimately lead to kidney failure. Our study, therefore, identifies P2Y_14_ as a potential therapeutic target for the prevention or treatment of sterile inflammation (and potentially pathogen-associated inflammation) in the kidney. In this context, we are currently examining whether the UDP-glucose/P2Y_14_ signaling pathway is involved in the physiology and pathophysiology of the human kidney.
